# Pivotal role of BCL11B in the immune, hematopoietic and nervous systems: a review of the BCL11B-associated phenotypes from the genetic perspective

**DOI:** 10.1038/s41435-024-00263-w

**Published:** 2024-03-12

**Authors:** José María García-Aznar, Sara Alonso Alvarez, Teresa Bernal del Castillo

**Affiliations:** 1 Healthincode, A Coruña, Spain; 2 Universitary Institute of Oncology of Principado de Asturias (IUOPA), Oviedo, Spain; 3 Health Research Institute of Principado de Asturias, Oviedo, Spain; 4 Hematology Department, Hospital Universitario Clínico de Asturias, Oviedo, Spain

**Keywords:** Disease genetics, Primary immunodeficiency disorders

## Abstract

The transcription factor BCL11B plays an essential role in the development of central nervous system and T cell differentiation by regulating the expression of numerous genes involved in several pathways. Monoallelic defects in the BCL11B gene leading to loss-of-function are associated with a wide spectrum of phenotypes, including neurological disorders with or without immunological features and susceptibility to hematological malignancies. From the genetic point of view, the landscape of BCL11B mutations reported so far does not fully explain the genotype-phenotype correlation. In this review, we sought to compile the phenotypic and genotypic variables associated with previously reported mutations in this gene in order to provide a better understanding of the consequences of deleterious variants. We also highlight the importance of a careful evaluation of the mutation type, its location and the pattern of inheritance of the variants in order to assign the most accurate pathogenicity and actionability of the genetic findings.

## Introduction

Transcription factors are one of the most potent players in the regulation of gene expression. The B-cell lymphoma/leukemia 11B gene, BCL11B, is a zinc finger transcription factor first described in chicken in the 2000s by Avram et al. [1], who reported its pleiotropic functions, involving T-cell maturation, central nervous system development and craniofacial organization. As its paralog BCL11A, BCL11B it is expressed in the central nervous system, craniofacial tissues and lymphocytes, among other tissues [1, 2]. However, both genes differ in their disease-causing mechanisms. As an example, BCL11A has been found to be overexpressed in chronic lymphocytic leukemia, immunocytoma and Natural Killer/T-cell lymphoma, suggesting that gain-of-function is the molecular mechanism of pathogenicity [3]. By contrast, BCL11B haploinsufficiency is associated with T-cell Acute Lymphoblastic Leukemia (T-ALL) and neurological disorders. Curiously, haploinsufficiency of BCL11A is also responsible for the development of Días-Logan syndrome, a neurological disorder with intellectual disability [4].

BCL11B is placed in the distal region of the chromosome 14, at band 14q32.2. In humans, alternative splicing of BCL11B transcripts results in four isoforms, which differ in the presence or absence of exon 3 and the length of the corresponding protein. The reference isoform, isoform 1 (NM_138576.4; NP_612808.1), contains 894 amino acids and is encoded by four exons, which are organized in six Zinc finger C2H2-type domains [5]. In vitro studies using directed mutagenesis have elucidated the molecular bases that regulate the function of BCL11B. In this regard, this gene seems to act as a homodimer, in a way that the zinc finger domains are essential to bind to consensus sites of a variety of genes, such as IL2 and CDKN1A, among others [6, 7]. The third and fourth zinc finger domains are involved in the binding to DNA, whereas the first, second, fifth and sixth zinc finger domains interact with the proteins MTA1/2, HDAC1/2 [8, 9]. BCL11B possesses activator and repressor properties. Hence, it promotes the transcription of IL2, cooperating with p300 coactivator, while it also inhibits the transcription of genes such as p57, p21 and SKP2, when it interacts with the nucleosome remodeling and histone deacetylation complex (NuRD) [7, 10]. This explains why loss of function (LoF) of this integral factor leads to the development of a wide spectrum of diseases.

To understand the genotype-phenotype correlation, we need to understand the function of this gene in specific organs and systems. In this review we have carried out a review covering the different functions and related phenotypes of BCL11B, describing the landscape of genetic variants and providing keys for the interpretation of the genetic defects in this gene.

### BCL11B and the immune system

The process of BCL11B activation starts when NOTCH1/IL7R signaling promotes the normal activation of BCL11B during thymocyte differentiation with the coordinated action of the transcription factors CSL, TCF-1 and GATA3, all of which are upregulated by NOTCH-IL7. Once it is activated, the expression of the gene is sustained through the action of RUNX factors [11, 12].

BCL11B is required at several steps of T-cell precursor differentiation. In the double-negative stage 2 (DN2) T-cell precursors, BCL11B promotes the transition into T-cells α/β T-Cell Receptor (TCR) through V_β_D_β_ rearrangement. It suppresses Natural Killer and dendritic potential in DN2 and DN3 stages and blocks TCR-independent stemness. Consistent with the above, loss of BCL11B function disrupts the development of T-cell α/β TCR in favor to γ/δ TCR [10]. DN2 T-cells in which BCL11B have been deleted acquire NK cell properties [13]. Knockout BCL11B−/− mice experiment severe pre-T-cell apoptosis in the thymus, which prevents α/β recombination of VDJ rearrangements at DN stage [14]. Knockout-murine models with complete LoF of BCL11B develop T-cell malignancies, as a consequence of dysregulated T-cell maturation [15]. In this sense, BCL11B acts as an haploinsufficient tumor suppressor, collaborating in the oncogenic process with other genetic events [16]. Recently, it has been described a dual role of BCL11B in oncogenesis in such a way that, when overexpressed, it promotes the survival of cancer cells by avoiding cancer cell damage and cooperating with RAS oncogene in transformation. On the other side, when it is inactivated, its capacity to stimulate several excision repair enzymes is lost, explaining its role as haploinsufficient tumor suppressor gene [17].

BCL11B exerts a crucial role in the proliferative response of peripheral CD8+ T-cells in response to viruses, intracellular bacteria and tumors. It regulates CD8 coreceptor gene expression through association with Ei8 and other specific enhancers. Since the coreceptor amplifies TCR signaling, BCL11B defects are associated to reduced antigen-specific clonal expansion, proliferation and cytolytic function [18].

BCL11B is also linked to autoimmune diseases. This transcription factors induces FOXP3 gene expression in CD4+ cells in response to TGFβ1, by binding to the non-coding sequence. Consequently, defects in BCL11B lead to reduced FOXP3 gene expression and induce Treg generation from conventional CD4+ T-cells. In turn, these Treg cells show reduced expression of IL-10 gene (which is known to play an important role in Treg suppression) and increased levels of proinflammatory cytokines [19].

### BCLL11B and the nervous system

Embryological studies in murine and human models have revealed that BCL11B is expressed in the neocortex, hippocampus, vomeronasal organ and basal ganglia where is required for the correct development and function of axonal projection of corticospinal motor neurons [20]. It has a critical role in the neurogenesis of the hippocampus, where it promotes proliferation of progenitor cells and regulates cell differentiation. The absence of BCL11B leads to profound impairment of hippocampus and motoneurons. In adults, BCL11B is controls the differentiation and survival of mature neurons by up- and down-regulating target genes such as DSP, CALB1, HTR2C and TACR3 [21].

### BCL11B in skin development and wound healing

Murine models have revealed that BCL11B controls the keratinocyte proliferation and late epidermal differentiation events leading to the development of the epidermal permeability barrier. This effect is mediated by the regulation of specific transcription factors involved in epidermal homeostasis [22].

Increased expression of BCL11B has been described in the basal and suprabasal layers of the epidermis of patients with atopic dermatitis, contributing to the hyperproliferative state which characterizes this condition [23]. Defects in BCL11B in keratinocytes lead to altered keratinocyte activation and reduced proliferation through EGFR down regulation. In addition, NFATC1 hyperexpression takes place in BCL111B mutants, suppressing stem cell activation and delaying wound healing [24]. Consequently, the absence of BCL11B in murine models revealed reduced epithelial thickness in the epidermis and aberrant polarization in the epithelial cells, which also have consequences for tooth formation and leads to accelerated ossification of skull sutures [22, 25].

**Fig. 1:**
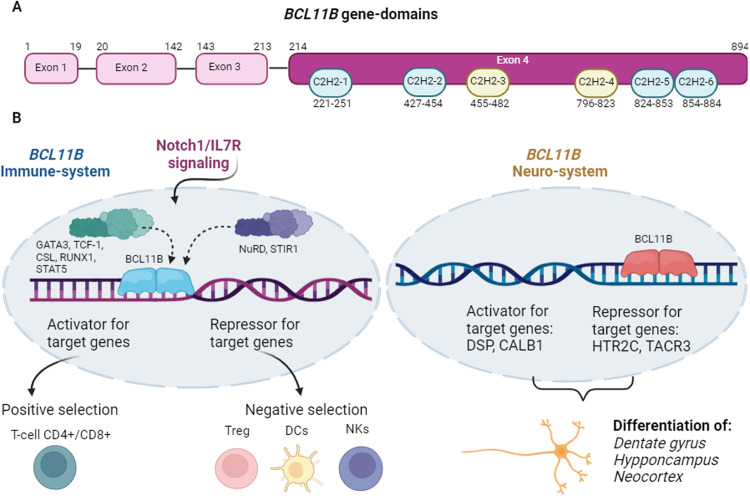
Representation of the gene structure, domains and molecular pathwaysinvolved in the differentiation of neurogenesis and lymphocytes. **A** Representation ofthe gene structure and domain of BCL11B. The positions of the start and end ofthe amino acid positions of the exons and domains were captured from uniprot database (www.uniprot.org). **B** Representationof the molecular pathways involved in the differentiation of neurogenesis (right) and lymphocytes (left).

The gene structure and molecular pathways involving immune and neurologic systems regulated by BCL11B are depicted in Fig. 1.

#### BCL11B-associated phenotypes

Considering that BCL11B is expressed in multiple organs, tissues and cells, and its implications in a wide range of cellular processes, it is not surprising that its loss of function results in a complex variability of disorders. However, the correlation between genotype and phenotype has not been completely understood. In an attempt to classify these phenotypes, several authors have described the following syndromic disorders: immunodeficiency, hematologic malignancies and neurological disorders.

### Primary immunodeficiency

Severe Combined Immunodeficiency (SCID) associated with monoallelic LoF mutations in BCL11B was first described in 2016 by Punwani et al. [6]. This syndrome, which begins in the neonatal period, is characterized by T-cell lymphopenia at the expense of CD4+ T cells, impaired T-cell response, and predisposition to T-ALL. It may be accompanied by dysfunction of B-cells, with defects in the switch from naïve to memory B-cells [6, 26]. Other clinical manifestations include dysmorphic features, neurodevelopmental abnormalities and a profound immunological dysregulation expressed as allergy, asthma, eczema, eosinophilia and severe atopy [27]. Most of the reported variants were of germline origin, occurring de novo or being inherited from an affected parent, from which is inferred a dominant inheritance pattern with complete penetrance.

### Hematological malignancies

Haploinsufficiency of BCL11B contributes to impaired T-cell differentiation and clonal expansion, leading to the development of T-ALL [28]. Truncating and missense variants in this gene have been reported in 9% of T-ALL patients, with enrichment in mutations located in exon 4 [8]. BCL11B silencing has also been observed as a consequence of structural variants such inv(14) in T-ALL [29]. Interestingly, chromosomal rearrangements involving BCL11B, have also been found in acute myeloid leukemia (AML) [30].

Variations in BCL11B expression have also been described in T-ALL, with opposite results. On the one hand it has been demonstrated that overexpression triggers apoptosis resistance [7]. By contrast, low expression was associated with lower survival in a homogeneously treated population of 169 T-ALL patients [31].

Finally, rare variants in BCL11B have been detected in T-ALL patients accompanying other relevant genetic defects. In a previous work, we found a missense variant in BCL11B located in a C2H2-type domain in a T-ALL patient accompanied by a SIL-TAL1 rearrangement and deletions in CDKN2A/B [32]. In the same report, one patient harbored a deleterious variant in BCL11B with an actionable FLT3-internal tandem duplication.

In summary, the wide range of potential BCL11B alterations highlights the central role of this gene in T-ALL leukemogenesis.

### Neurological disorder

SCID syndrome caused by BCL11B loss-of-function also presents with severe neurodevelopmental abnormalities and craniofacial dysmorphism appearing at neonatal age. The most common neurological symptoms include intellectual disability, speech impairment, autistic features and motor delay. Although some patients may not present obvious immunological abnormalities, intellectual disability is present in 100% of them [33]. The expression of dysmorphic features is variable and may include myopathic facial appearance, thinning eyebrows, small palpebral fissures, hypertelorism, prominent nose, long philtrum, thin upper lip, ocular and dental abnormalities. Other rare symptoms reported are dysgenesis of corpus callosum, ventriculomegaly, spasticity of lower limbs and schizophrenia [34, 35].

### Craniosynostosis

BCL11B is expressed in cranial osteogenic and sutural mesenchyme and plays an essential role in the regulation of suture patency. In mice, it has been demonstrated that germline deletion of BCL11B leads to craniofacial synostosis, a disorder defined by the premature fusion of cranial sutures [36]. In humans, both de novo and inherited mutations result in complex syndromes combining craniosynostosis with immunodeficiency [6], dysmorphic features, or neurodevelopmental delay [37–39]. The mutations in these patients act by disrupting the binding of the protein to its target DNA, which results in haploinsufficiency [38, 39] or redirecting the protein binding to novel sites [6].

## Review of literature from genetic perspective

While previous works have described series of patients with predominant neurological or neoplastic manifestations [16, 26], in our work we have reviewed the whole range of manifestations that may arise as a consequence of genetic defects in BCL11B. Likewise, we have tried to show that a close relationship exists between the type of mutations, the pattern of inheritance and the phenotype. Finally, we have extended the review of the role of BCL11B beyond the immune system [2], offering an overview of its role in the development of skin and nervous system.

We systematically analyzed PubMed, Medline and EMBASE for all publications describing BCL11B mutations using the keywords and search terms “BCL11B”; “genetic mutation”/”variant”. Until August 2022, 143 references were retrieved. Publications were included if they met these criteria: 1) case reports, case series, conferences abstracts and retrospective studies; 2) description of immunodeficiency or neurological disorder or craniosynostosis or leukemia; 3) The reported mutations were considered by the authors as disease-causing or potentially disease-causing. Table 1 shows the publications selected, together with the mutations described in each one and the clinical phenotype.

**Table 1:** Summary of the publications containing information about BCL11B mutations, number of affected patients and clinical manifestations.

Reference	Number of patients	Mutations	Mutation type	Number of exon	In silico prediction	Clinical manifestations
Goos et al. [37]	1	p.Arg3Ser	Nonsynonymous	1	Deleterious	Craniosynostosis
Liu et al. [52]	19	p.Leu30fs* p.Asp33fs*6 p.Leu36_Glu37fs* p.Thr120fs* p.Cys432Arg p.Arg447Cys p.Thr450fs* p.Glu452Lys p.Pro454Thr p.Tyr455Asn p.Tyr455HIs p.Tyr455Cys p.Ser465Leu p.Gln466His p.Lys469Thr p.Arg472Cys p.Arg472His p.Lys475Glu p.Gln835His	Frameshift Frameshift Frameshift Frameshift Nonsynonymous Nonsynonymous Frameshift Nonsynonymous Nonsynonymous Nonsynonymous Nonsynonymous Nonsynonymous Nonsynonymous Nonsynonymous Nonsynonymous Nonsynonymous Nonsynonymous Nonsynonymous Nonsynonymous	2 2 2 2 4 4 4 4 4 4 4 4 4 4 4 4 4 4 4	Truncating Truncating Truncating Truncating Deleterious Deleterious Truncating Deleterious Deleterious Deleterious Deleterious Deleterious Deleterious Deleterious Deleterious Deleterious Deleterious Deleterious Deleterious	T-ALL
Che et al. [49]	4	c.427+1G>A (x3) p.Glu821Glyfs∗28	Splicing Frameshift	2 4	Truncating Truncating	Neurodevelopmental disorders
Lessel et al. [26]	10	p.Cys81Leufs*76 p.Tyr455* p.Glu499* p.Thr502Hisfs*15 p.Asp534Thrfs*29 p.Arg581Alafs*45 p.Gly649Alafs*67 p.Asn807Lys p.Gly820Alafs*27 p.Ala891Profs*67	Frameshift Nonsense Nonsense Frameshift Frameshift Frameshift Frameshift Nonsynonymous Frameshift Frameshift	2 4 4 4 4 4 4 4 4 4	Truncating Truncating Truncating Truncating Truncating Truncating Truncating Deleterious Truncating Truncating	Neurodevelopmental disorder with severe combined immunodeficiency
García-Aznar et al. [32]	3	p.His126Tyr p.Thr450Met p.Gly581Asn	Nonsynonymous Nonsynonymous Nonsynonymous	2 4 4	Deleterious Deleterious Tolerated	T-ALL
Neumann et al. [53]	6	p.Gln167His (p.Gln238His) p.Tyr384* p.Thr450Met (p.Thr379Met) p.Arg472His (p.Arg401His) p.Ala834Thr (p.Ala763Thr) p.Gln848Arg (p.Gln777Arg)	Nonsynonymous Frameshift Nonsynonymous Nonsynonymous Nonsynonymous Nonsynonymous	3 4 4 4 4 4	Deleterious Truncating Deleterious Deleterious Deleterious Deleterious	T-ALL
Yang et al. [51]	1	p. Ser398Glnfs∗117	Frameshift	4	Truncating	Neurodevelopmental disorder with severe combined immunodeficiency
Gaillard et al. [54]	4	p.Pro422Leu p.Gly582Ser p.Gly667Glu p.Pro673Arg	Nonsynonymous Nonsynonymous Nonsynonymous Nonsynonymous	4 4 4 4	Deleterious Deleterious Deleterious Deleterious	Craniosynostosis
Punwani et al. [6]	1	p. Asn441Lys	Nonsynonymous	4	Deleterious	+
Gutierrez et al. [16]	5	p.His445Tyr p.Arg447His p.His479Tyr p.Gly596Ser p.Gly847Arg	Nonsynonymous Nonsynonymous Nonsynonymous Nonsynonymous Nonsynonymous	4 4 4 4 4	Deleterious Deleterious Deleterious Deleterious Deleterious	T-ALL
Seki et al. [55]	1	p.Thr450Met	Nonsynonymous	4	Deleterious	T-ALL
Yan et al. [50]	1	c.1887_c.1893delCGGCGGG; p.Gly630Thrfs*91	Frameshift	4	Truncating	Neurodevelopmental disorder
Prasad et al. [34]	2	p.Gly649Alafs*67 p.Asn807Lys	Frameshift Nonsynonymous	4	Truncating Deleterious	Neurodevelopmental disorder
Qiao et al. [33]	1	p.Thr730Thrfs*151	Frameshift	4	Truncating	Neurodevelopmental disorder
Zhao et al. [38]	1	p.Gly783Alafs*24	Frameshift	4	Truncating	Neurodevelopmental disorder
Lu et al. [27]	2	p.Cys826Tyr	Nonsynonymous	4	Deleterious	Neurodevelopmental disorder with severe combined immunodeficiency

fs* = frameshift mutation; * = stop codon (nonsense variant).

The genotypic and phenotypic information was collected and organized according to the following categories: demographics (sex, age of onset, age at study), clinical data (clinical diagnosis and phenotypes), variant significance (relevant variants, inheritance, allele frequency, in silico prediction, functional effect and classification of the pathogenicity). All this information is extensively detailed in Supplementary Table 2.

Clinical data were grouped into one of these clinical entities: intellectual developmental disorder with dysmorphic facies, craniosynostosis, T-cell abnormalities and T-ALL (Table 2). This information was completed with the in-silico predictions from six different sources and the classification of the pathogenicity according to American College of Molecular Genetics criteria (ACMG) [40]. The computational tools allowed us to support a predicted deleterious/tolerated effect for missense mutations based on alignment of protein sequence, conservation, probability of mutations and models of neural network. The following bioinformatic predictors were used: Sorting Intolerant from Tolerant (SIFT) [41]; Polymorphism Phenotyping v2 (PolyPhen-2) [42], MutationTaster [43], Combined Annotation Dependent Depletion (CADD) [44], DANN score (based on a deep neural network) [45] and Functional Analysis through Hidden Markov Models (FATHMM) [46]. Each tool provides a score that indicates the impact or pathogenicity of the variants on the function of a human protein. The score ranges of the different tools are shown in Supplementary Table 2. In order to classify somatic mutations, the guidelines of Clinical Genome Resource (ClinGen), Cancer Genomics Consortium (CGC), and Variant Interpretation for Cancer Consortium (VICC) [47] were used. Finally, variants identified in patients diagnosed with T-ALL in which information regarding their somatic status was missing were searched in the COSMIC database [48]. The variants were annotated based on isoform 1 (NM_138576.4; NP_612808.1).

**Table 2:** Clinical features of patients included in this review. Distribution of BCL11B-truncating and missesense variants.

Phenotype	Individuals with truncating mutations (*N* = 24)	Individuals with missense mutations (*N* = 38)
Neurological disorders with or without immunological defects	18/24 (75%)	10/38 (26%)
Intellectual developmental disorder with dysmorphic facies, speech delay, and T-cell abnormalities	4/24 (17%)	3/38 (8%)
Isolated neurodevelopmental delay	1/24 (15%)	0
Neurodevelopmental disorder with craniosynostosis	1/24 (4%)	5/38 (13%)
Neurodevelopmental disorder with defects of immunotolerance	7/24 (29%)	3/38 (8%)
Neurodevelopmental disorder with facial dysmorphism without immunological features	4/24 (17%)	6/38 (16%)
T-ALL	6/24 (25%)	28/38 (74%)

The possible phenotypes resulting from BCL11B defects are divided into two large groups: neurological disorders with or without immunological defects and T-ALL (bold). Neurological disorders with or without immunological defects are further broken into five categories (rows 3–7). Whitin each group of clinical disorder a further division has been stablished according to the number of individuals with truncating mutations (second column) or missense mutations (third column).

### Review of variants in BCL11B according to mutation-type (truncating versus missense)

At least 54 different disease-associated mutations have been reported in the BCL11B gene. Most of the reported mutations are located in exon 4, with a predominance of nonsynonymous (34/45) over frameshift (14/45) and splicing (1/45) or nonsense (2/45) variants. Seven mutations have been described, in exon 2, four of them frameshift, one deleterious and one nonsynonymous. Only one mutation has been described in exon one and other in exon three. Details regarding these mutations are shown in Table 1 and Fig. 1.

The mutations were identified in 62 patients, of whom 24 harbored truncating mutations and 38 missense variants. Since it has been demonstrated that monoallelic LoF variants are responsible for a BCL11B-related phenotype, all truncating mutations found in the sequence of BCL11B have been classified as pathogenic or likely pathogenic. Only four functional studies have proved loss of function of missense mutations. In the remaining, missense variants, either functional studies or robust logarithm of the odds would be required to classify them as pathogenic.

### Truncating variants

#### Exon 2

A frameshift variant located in exon 2, p.Cys81Leufs*76, was predicted to suffer nonsense-mediated mRNA decay (NMD) and non-stop mRNA decay (NMD), resulting in haploinsufficiency of the protein [26]. The variant p.Ala891Profs*106, placed in the last coding residue, was predicted to extend the protein translation beyond the last coding residue, suffering non-stop mRNA decay and haploinsufficiency of the protein, too. In the same study, seven truncating variants located in exon 4 (p.Tyr455*, p.Glu499*, p.Thr502Hisfs*15, p.Arg518Alafs*45, p.Asp534Thrfs*29, p.Gly649Alafs*67 and p.Gly820Alafs*27) were assumed to escape this mechanism of mRNA degradation. These changes are highly likely to result in proteins lacking the last Zinc-finger domains. However, only the p.Gly820Alafs*27 variant was tested to assess the stability of the allele carrying the mutation. Immunohistological staining showed that no stable cDNA was expressed in the mutant compared to the wild-type allele. Real-time PCR was not done in the other variants to confirm the predicted haploinsufficiency effect.

The only splicing variant presented in this review, c.427+1G>A in exon 2 (observed in three patients from same family), produced the exon skipping during the splicing process, leading to the introduction of a premature stop codon at p.Val48Glyfs∗14, before C2H2-1 finger domain. The aberrant transcript caused by this variant was supposed to be degraded by NMD, but western blotting and immunofluorescence assays revealed normal expression and no significant changes in cell morphology or localization [49], suggesting the existence of post-transcriptional regulatory mechanisms affecting protein function as the disease-causing mechanism of the mutation.

#### Exon 4

According to the algorithm of the computational tool AlphaFold, the frameshift mutation p.Gly783Alafs*24, placed the stop codon in the C2H2-4 domain in exon 4. It was also predicted to escape NMD. This truncated protein would lack the last three C2H2 zinc-finger domains and hence affect the protein´s function in binding to its target DNA. However, the authors recommend further experiments to elucidate the true mechanisms of action of the variant [38].

Regarding the insertion c.2461_2462ins (p.Glu821Glyfs∗28), located in the exon 4, the results observed in the qPCR assay showed no difference in the level of mRNA between mutant and wild-type transfected cells. Therefore, the scape of this variant form NMD was confirmed. This variant loses the last two C-terminal DNA binding Zinc-finger domains, probably resulting in a truncated protein with a pathogenic mechanism of haploinsufficiency [50].

The variants p.Ser398Glnfs∗117, p.Gly630Thrfs*91 and p.Thr730Thrfs*151 were described in de novo case reports [33, 50, 51]. According to the position of the premature stop codon, these three variants were expected to escape nonsense‐mediated mRNA decay. This would lead to a truncating protein, conserving the first zinc finger domain in the variant p.Ser398Glnfs*117 and the first, second and third finger domains in the case of the variants p.Gly630Thrfs*91 and p.Thr730Thrfs*151.

Six truncating variants (p.Leu30fs*, p.Asp33fs*, p.Leu36_Glu37fs*, p.Thr120fs* located in exon 2 and p.Tyr384*, p.Thr450fs* located in exon 4) were identified only in patients with T-ALL; Surprisingly, in none of them functional studies were performed and neither had confirmed zygosity [52, 53]. Nevertheless, p.Asp33fs* and p.Leu36_Glu37fs* were reported in COSMIC database as somatic mutations in specimens with T-ALL. Although the molecular mechanism of pathogenicity was not studied, the variants p.Thr120fs*, p.Tyr384*, p.Thr450fs* would scape NMD, while the rest will directly suffer NMD.

#### Missense variants

Thirty-eight patients harbored 33 unique non-synonymous mutations located throughout the four exons. The most frequent diagnosis was T-ALL in 28 patients, whereas neurological disorders were present in 10 of the 38 patients. Among the latter, 4 showed concomitant immunological disorders. Germline origin was confirmed in patients with neurological disorders.

Amino acid substitutions secondary to missense mutation were the most frequent defect in T-ALL. Regarding these missense variants, all but 3 SNVs had a maximum allele frequency of <0.005%, suggesting that this gene is very conserved, in line with the function of transcription factors which maintain high fidelity in their aminoacidic sequence especially in DNA-binding motifs.

#### Exon 1

The p.Arg3Ser variant was the only reported mutation located within the first exon of BCL11B (Table 1 and Fig. 1). This missense variant occurred de novo in a patient with seizures, dysmorphic features and craniosynostosis. No immunological alterations were observed. The variant was absent in the control population and 5 out of 6 bioinformatics tools predicted a deleterious effect. The functional study of p.Arg3Ser in HEK293T-cells showed normal expression and localization of BCL11B mutants, but inability of the protein to interact both with the NuRD and PRC2 complexes. The substitution of Arg3 changed the charge of the “RRKQxxP” motif, generating a novel potential site for post-translational modification suggesting alternative positions at nearby residues [35]. Specifically, this variant causes a change from an amino acid with a basic side chain (arginine) to an amino acid with a neutral polar side chain (serine). Finally, a mouse model for BCL11B p.Arg3Ser mutants showed coronal suture craniosynostosis in both heterozygotes and homozygotes, with a less severe and more variable phenotype in heterozygotes.

#### Exon 2

The p.His126Tyr variant, located in exon 2, was reported in a patient with ETP-ALL as a rare finding together with a pathogenic mutation in the FLT3 gene. This variant presented an alternative allele frequency of 50%, suggesting germline origin. In fact, this change had 9 heterozygotes in the general population. However, since it has not been confirmed in non-hematological tissues, the germline origin should not be definitely considered [32].

#### Exon 3

A non-described variant, p.Gln238His (p.Gln167His) located in exon 3, was detected in an adult patient with T-ALL. No functional studies were performed [53].

#### Exon 4

Germline mutations p.Pro422Leu, p.Gly582Ser, p.Gly667Glu, and p.Pro673Arg were found in patients with craniosynostosis and congenital diaphragmatic hernia. In all cases parents were unaffected, suggesting incomplete penetrance [54]. They present in a very low frequency in general population and are located in a hotspot region for pathogenic mutations. The in-silico predictor prognosed a likely tolerate effect. Therefore, they should be considered variants of unknown significance. Further functional studies would be required to clarify the pathogenicity of the mutations.

Numerous missense mutations situated in exon 4 (p.Cys432Arg, p.Arg447Cys, p.Glu452Lys, p.Pro454Thr, p.Tyr455Asn, p.Tyr455His, p.Tyr455Cys, p.Ser465Leu, p.Gln466His, p.Lys469Thr, p.Arg472Cys, p.Arg472His, p.Lys475Glu, p.Gly581Asn, p.Gly596Ser, p.Ala834Thr, p.Gln835His, p.Gly847Arg and p.Gln848Arg) have been identified in pediatric or young patients diagnosed with T-ALL, with a deleterious effect according to computational results and a very low allele frequency or even absence in the general population [16, 32, 52]. All of them were concentrated in the C2H2-2/3 and 4 domains, which are considered a hot-spot for pathogenic mutations in BCL11B.

Using high-resolution crystal structure of the zinc finger domains and based on models with structural homology of canonical DNA binding of BCL11B, Gutierrez et al. [16] evaluated four missense variants (p.His445Tyr, p.Arg447His, p.Arg472His, p.His479Tyr) located withing this hot-spot in exon 4. The results showed that the affected residues disrupted DNA binding by destabilization of the conserved amino acids. Although a functional study of p.Thr450Met was not performed, it was recurrently found in patients with T-ALL with poor prognosis [32, 52, 55]. Considering its low prevalence in the general population and its zygosity compatible with heterozygous state (allele frequency close to 50%), it might be argued that this change is a rare germline variant with a high potential of having a deleterious effect on BCL11B. The variants p.Asn807Lys [26] and p.Cys826Tyr [21] require special attention because of their publication in two unrelated patients as de novo. Both are located in exon 4 and affect domains C2H2-4 and C2H2-5, respectively. The p.Asn807Lys variant was predicted to affect interaction between specific residues of the C2H2-4 domain and DNA. The p.Cys826Tyr variant was evaluated by structural modeling, using the crystal structure of the related transcription factor BCL11A. This model revealed that the change could perturb the coordination of zinc ions, thereby affecting protein interaction [27]. A functional study in a zebrafish model showed that mutant T-cells were unable to differentiate to terminal states. Patients harboring these mutations also showed neurological disorders with dysmorphic features and T-cell disturbances such as low Treg counts and hyper IgE syndrome.

**Fig. 2:**
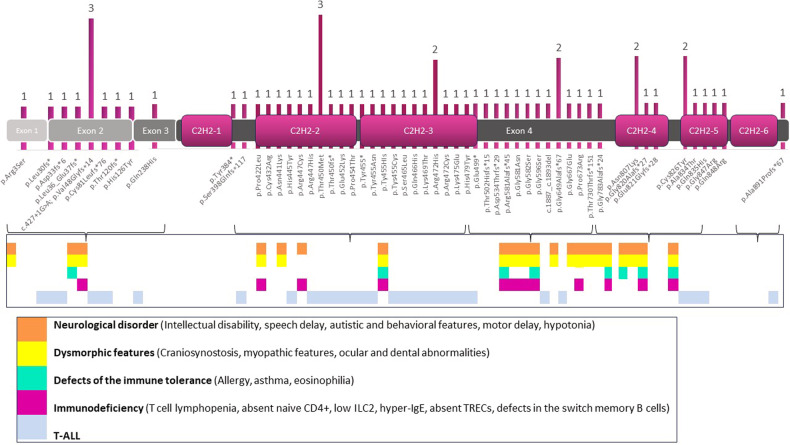
Representation of reported mutations in BCL11B and clinical features of patients. On the top of the figure, the reported variants in BCL11B and their location along gene structure are shown. The vertical bar represents the number of patients harboring each mutation. As can be noticed, there are variants identified in more than one patient: c.427+1G>A (3 patients), p.Thr450Met (3 patients), p.Gly649Alafs*67 (2 patients), p.Asn807Lys (2 patients), p.Cys826Tyr (2patients). On the bottom of the figure, the horizontal boxes show the clinical phenotypes driven by specific BCL11B mutations. Each color indicates specifically the clinical and laboratory alterations observed in the patients: orange indicates neurological disorders yellow indicates dysmorphic features; turquoise indicates defects in immune tolerance; pink indicates hematological malignancies; blue indicates immunodeficiency.

No mutations have been described in exons first and sixth Zinc finger domains. Figure 2 shows the mutations located along the BCL11B gene.

#### Genotype-phenotype correlation

The probability of being Loss-of-function-Intolerant (pLI) of BCL11B is 0.99, suggesting that this gene would not tolerate the presence of variants with LoF effects such as nonsense, frameshift and splicing [56–58]. In fact, LoF mutations in BCL11B cause two major clinical pictures that do not overlap: T-ALL and neurological disorders. Recurrent infections and immunological disturbances may be present in the last group.

From the review it might be suggested that a relationship between the phenotype and the mutation-type could be stablished; 28 out of 34 T-ALL patients harbored missense mutations compared to 10 out of 28 patients with neurological disorders who harbored missense mutations. In other words, missense mutations are responsible for the majority of T-ALL whereas truncating mutations predominate in patients with neurological disorders. The distribution of the type of mutations according to the clinical phenotype is shown in Table 2.

**Fig. 3:**
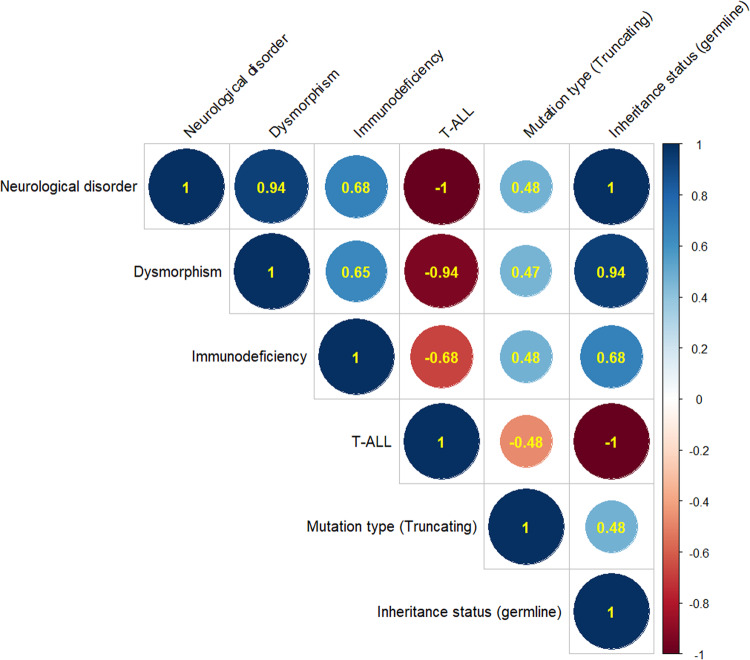
Genotype-Phenotype correlation in BCL11B. Representation of the correlation between type of mutation (truncating versus truncating), pattern of inheritance (germline versus somatic) and phenotype by using Pearson correlation coefficient. The coefficient ranges from -1 to 1. A positive correlation is marked in blue color, whereas the negative correlation is indicated in red color. The more intense is the color, the stronger the correlation.

In addition, we compared the correlation between genetic variables such as mutation type and inheritance status and phenotype by using the Pearson’s correlation coefficient. The results of this comparison are shown in Fig. 3 and shows the association between germline truncating mutations and neurological disorders and somatic missense mutations and T-ALL, respectively. More detailed information regarding the pattern of inheritance is shown in Supplementary Table 2.

## Discussion

In this review we have gathered information about mutations in BCL11B published to date. We have focused on potentially disease-causing mutations and extensively reviewed their molecular structure and their functional and clinical consequences. Some of these mutations, particularly truncating mutations, had been classified as mutations of uncertain significance according to ACMG criteria. However, after applying bioinformatic predictors, we suggest that most of them could be reclassified as pathogenic. Our review was also aimed to clarify the contexts that need to be considered when interpreting the consequences of the reported BCL11B variants. Specifically, the type of the mutations (truncating or missense), the location in the domains of the protein and the level of expression in the affected tissue and the pattern of inheritance (germline or somatic) must be considered.

In general, truncating mutations are associated to neurodevelopmental disorders with dysmorphic features and immunologic disturbances. Rare missense variants causing BCL11B haploinsufficiency are also responsible for some cases of neurological disorders. It is interesting that no missense mutations were reported in patients with neurological disorders in exons 2 and 3, and only two missense variants placed in these two exons were found in patients with craniosynostosis and T-ALL, which suggests that this region is not a hot-spot. On the other hand, missense mutations and deletions are associated with T-ALL. It may be hypothesized that such skewed distribution in the type of mutations related to the phenotype may be explained by the level of expression of BCL11B in the affected tissues and that epigenetic regulation of the expression is responsible for the differences. In line with this argument, hypomorphic missense mutations in BCL11B express variable features, different to the ones observed in truncating mutations.

Regarding the pattern of inheritance, it is striking that 100% of the disease-causing variants in BCL11B-neurological disorders were of germline origin, either de novo or inherited from an affected parent. In contrast, nearly all the missense variants detected in T-ALL patients were somatic or of unknown origin. An exception to this rule is a recently reported mutation, p.Thr450Met, considered of probable germline origin. This heritability of the disease has obvious clinical implications when selecting donor from siblings in case of performing hematopoietic stem cell transplant. The lack of information regarding zygosity from missense variants in patients with T-ALL constrains the possibility of estimate the genotype-phenotype correlation as well as the functional consequences of the variants. Finally, there were no reported patients with missense germline mutations with immunological abnormalities as the sole manifestation of the disease, i.e. without extra-immune manifestations. Something similar happened in other restricted phenotypes associated with LoF mutations in BCL11B, such is craniosynostosis without other symptoms or milder features.

Mutations causing immunological abnormalities such as low peripheral blood T lymphocyte counts, dysfunctional type 2 innate lymphoid cells and autoimmunity, do not share a common pattern, making it difficult to predict the effect of such mutations. The plasticity of the immune system could explain the modulation in the expressiveness of the BCL11B defects. In fact, the variability of the expression is comparable to that observed in other diseases caused by defects in transcription factors with a wide range of target genes, such as IKZF1 or GATA2 [59].

Although we have provided an extensive analysis about the genotype-phenotype correlation, there are several shortcomings that limit the generalizability of the findings. First, the reduced number of patients analyzed; second, the heterogeneity of their diseases and finally, the lack of functional studies, especially regarding missense mutations. Therefore, it is desirable that future reports include functional studies proving the pathogenicity of the variants.

## Conclusions

In summary, the relevance of the BCL11B gene is reflected in the wide range of disorders that arise as a consequence of its loss of function. In order to stablish the pathogenicity of the genetic variants of BCL11B and the heterogeneity of the clinical manifestations, several aspects must be considered, including the type of mutation, the position of premature stop codon, the inheritance status and the function of specific domains. The precise interpretation of the genetic defect in BCL11B may guide the development of targeted treatments, especially, in the context of genome editing. In this sense, attempts of restoring BCL11B function with antisense oligonucleotides in animal models of SCID have been made and pave the way for genome editing [6].Considering that BCL11B haploinsufficiency has leukemogenic effects, further studies should also address the regulation of BCL11B expression at the molecular level. Finally, functional data could elucidate the consequences of nonsynonymous variants in the BCL11B gene.

### Supplementary information

Supplementary Table 1.

Supplementary Table 2

Supplementary List_of_Abbreviations
