# Supplementary material for: Pivotal role of BCL11B in the immune, hematopoietic and nervous systems: a review of the BCL11B-associated phenotypes from the genetic perspective
Source: Genes Immun. 2024 Mar 12;25(3):232–41. doi: 10.1038/s41435-024-00263-w (PMC11178493; doi:10.1038/s41435-024-00263-w)
Supplement: Supplementary file 1 — Supplementary Table 1. [file 41435_2024_263_MOESM1_ESM.docx]

**Supplementary Table 1.** Scores of the bioinformatic tools used for predicting pathogenicity.

| **Bioinformatic tool** | **Score range** | **Prediction** |
| --- | --- | --- |
| **SIFT** | 0.0 (deleterious)  to  1.0 (tolerated) | - **0.0 to 0.05.** Variants with scores in this range are considered deleterious. Variants with scores closer to 0.0 are more confidently predicted to be deleterious. - **0.05 to 1.0.** Variants with scores in this range are predicted to be tolerated (benign). Variants with scores very close to 1.0 are more confidently predicted to be tolerated. |
| **PolyPhen-2** | 0.0 (benign)  to  1.0 (deleterious) | - **<0,452.** Variants with scores below this value are considered likely benign. - **0,453 to 0,956**. Variants with scores between this range are considered possibly damaging. - **>0,957**. Variants with scores above this value are the most likely damaging. |
| **MutationTaster** | 0 to 1 | - Higher values indicate more deleterious cases. |
| **CADD** | 1 to 99  (normalized values) | - Higher values indicate more deleterious cases. The variants with values >20 are considered deleterious. |
| **DANN** | 0 to 1 | - Higher values indicate more deleterious cases |
| **FATHMM** | -16.13 to 10.64 | - Smaller scores are more likely to be deleterious. Scores below -1.5 are considered as deleterious. |
