# Supplementary material for: Pivotal role of BCL11B in the immune, hematopoietic and nervous systems: a review of the BCL11B-associated phenotypes from the genetic perspective
Source: Genes Immun. 2024 Mar 12;25(3):232–41. doi: 10.1038/s41435-024-00263-w (PMC11178493; doi:10.1038/s41435-024-00263-w)
Supplement: Supplementary file 3 — Supplementary List_of_Abbreviations [file 41435_2024_263_MOESM3_ESM.docx]

**Abbreviations**

**(in alphabetical order)**

α /β: Alpha/Beta

AML: Acute Myeloid Leukemia

ACMG: American College of Medical Genetics

BCL11B: B-cell lymphoma/leukemia 11B

BCL11A: B-cell lymphoma/leukemia 11A

CADD: Combined Annotation Dependent Depletion PolyPhen2:

CALB1:Calbindina 1.

CDKI1A: Cyclin dependent kinase inhibitor 1A

ClinGen: Clinical Genome Resource CLS: CBF1, Suppressor of Hairless

COSMIC: Catalog of Somatic Mutations in Cancer

DANN: Deep Annotation based on a deep Neural Network

DSP: Desmoplakin

EGFR: Epidermal Growth Factor Receptor

ETP-ALL: early T precursor ALL

FATHMM: Functional Analysis through Hidden Markov Models

FLT3: FMS Related Receptor Tyrosine Kinase 3

FOXP3: Forkhead Box P3

GATA2: GATA binding protein 2

GATA3: Trans-acting T-cell Specific Transcription Factor 3, GATA binding protein 3

gnomAD: Genome Aggregation Database

HDAC1/2

HTR2C: 5-hydroxytryptamine receptor 2C

IKF1: IKAROS Family Zinc Finger 1

IL2: Interleukin 2

IL7R: Interleukin 7 Receptor

LoF: Loss of Function

MTA1/2: Metastasis Associated Family Member Proteins 1/2

NFATC1: Nuclear Factor of Activated T cells

NMD: nonsense-mediated mRNA decay

NuRD: Histone deacetylation complex

NOTCH: Neurogenic locus notch homolog

RUNX1: RUNX Family Transcription Factor 1

SCID: Severe Combined Immunodeficiency

SIFT: Sorting Intolerant from Tolerant

STAT5a: Signal transducer and activator of transcription 5A

SKP2: S-Phase Kinase Associated Protein-2

TACR3: Tachykinin Receptor

T-ALL: T-cell Acute Lymphoblastic Leukemia

TCF: T Cell Factor

TCR: T Cell Receptor

TGFβ1: Transforming Growth Factor 1

Treg: T regulatory

PolyPhen-2: Polymorphism Phenotyping v2

PRC2: policomb repressive complex 2

VICC: Variant Interpretation for Cancer Consortium
